# Paradigm shift in science with tackling global challenges

**DOI:** 10.1093/nsr/nwz155

**Published:** 2019-10-22

**Authors:** Jinghai Li, Wenlai Huang

**Affiliations:** Institute of Process Engineering, Chinese Academy of Sciences, China

In recent years, the paradigm shift in science has become a hot topic in the global scientific community. Scientific problems unsolved with existing know-ledge or through traditional approaches can be found in all disciplines. On the other hand, more effective ways to meet global challenges, such as climate change, major diseases, natural disasters, and governance of social and economic systems, are still to be explored. Unfortunately it remains paralleled discussion on these two aspects, with the former involving a more generic view of the science development trend—the paradigm shift, while the latter concerning specific real-world problems to be solved.

What could be the bridge that connects these two aspects? We believe the following changes are highly important: firstly, research *contents* are being extended from equilibrium static states to dynamic structures and from local phenomena to system behavior; secondly, research *methods* have gradually shifted from qualitative analysis to quantitative prediction, from single discipline-based to transdisciplinarity-oriented, and from data processing to artificial intelligence; thirdly, research *domains* are moving from fragmented knowledge to integrated knowledge systems, from traditional theories to complexity sciences, from detail-focused to multiscale-associated, and from multilevel discipline-based study to the pursuit of universal principles. Additionally, it is very important for scientists to deduce common knowledge, even common principles, from disciplinary case studies in application-driven research. This propels the combination of the paradigm shift with transdisciplinarity and the specific solutions to major challenges. For example, traditional approaches based on coarse-graining and reductionism have to change to structural and systematic consideration, as discussed under the concept of mesoscience [1,2]. This new research strategy might be summarized as follows.

Both natural and artificial worlds are likely to be multilevel and each level is multiscale, containing the element scale, the system scale, and the in-between mesoscale [2]. For adjacent levels, the system scale at the lower level corresponds to the element scale at the upper level. Complexity usually emerges at the mesoscale when the system is operated in the mesoregime where at least two dominant mechanisms exist and they compromise with each other in the competition to realize their respective extremal tendencies. Therefore, in tackling complex issues, it is desirable to identify the involved levels first. At each level, it is necessary then to identify its element scale, system scale, and mesoscale. Subsequently, the mesoregime should be determined, where complex structures prevail (featuring spatiotemporal heterogeneity). Next comes the key step: the resolution of the multiple dominant mechanisms coexisting in the mesoregime under the specified internal and external conditions. Meanwhile, regime transitions can be defined by analysing the specified conditions and their influence on the system. The final step is to solve the multi-objective (variational or optimization) problem. Additionally, when multiple levels are involved, it is necessary to realize their correlations (see [2] for details).

Such a methodology, if adopted in research activities, could enhance the paradigm shift in science and lead to major breakthroughs. To be more specific, we would like to propose the following categories of issues in a variety of fields for further discussion.

## COLLECTIVE EFFECTS OF CHARGE CARRIERS

Room-temperature superconductivity, high-density energy storage, artificial photosynthesis, photovoltaics, thermo-electricity, and semiconductivity are the frontier issues that deserve intensive research in physics, chemistry, biology, and materials science. The common difficulty in this category is to understand relevant mechanisms governing carrier motion or transfer and to reveal the common scientific principles underlying these phenomena. These issues, probably engaged in the collective effects of charge carriers in different systems under different conditions, would vary with specific modes of carrier motion in specific systems and conditions, and the perspective of complex systems should be adopted in research, as discussed in [1].

## MULTILEVEL MATERIAL STRUCTURES

An object, man-made or natural at different levels, is composed of hierarchical elements (also systems at the adjacent levels) under certain circumstances. Therefore, the study in materials science needs to be hierarchical and to focus on the collective effects and system attributes of the elements at each level. In other words, it needs to clarify the changes in the environmental conditions of elements and systems during the formation of materials and to reveal the laws governing the collective behaviors of various elements at relevant levels. In particular, the changes in material structures triggered by system variations and the interdependence between levels are important.

## MULTILEVEL DYNAMIC CHANGES AND REGULATION IN MATERIALS PROCESSING AND SERVICE

As the core of all industrial processes, materials processing and service provide energy, devices, tools, and other necessities for human beings. It is also common in the natural environments where human beings live, and the key to the utilization of natural resources and prevention of disasters. How to perceive the dynamic structural changes in the processing and service of materials at all levels in order to optimize the performance of the products or enhance understanding of nature is always the core scientific issue underlying various types of manufacturing and relevant disciplines of natural sciences. Unlike the material structures elaborated in the above category, the external effects introduced by different modes of actions, such as mechanical, electrical, physical, and chemical treatments, often become the dominant factors. From laboratory processing to mass production, the complexity of the interaction between materials in different ways is always a big challenge. The evolutionary laws of various types of dynamic structures and their effects on product performance should also be studied along with the interaction between elements, system behavior, environmental conditions, dominant mechanisms, and other aspects of various levels of systems and elements.

## DYNAMIC EVOLUTION, INTERDEPENDENCE, AND REGULATION OF SUBSYSTEMS AT DIFFERENT LEVELS OF LIFE SYSTEMS

Apart from the genetic information, life systems share the same multilevel characteristics and similar laws of dynamic evolution with material systems. The dynamic changes of small molecules, biomacromolecules, cells, organs, and organisms at other levels in specific *in vivo* environments are of great significance to life and health. In particular, the structural and functional changes caused by the interaction of different levels and *in vivo* environmental changes should be the core of life systems. Issues to be addressed include: how to correctly identify different levels, whether immune mechanisms and metabolic processes can be explained from this mesoscience perspective, whether a spatiotemporal alternation between normality and lesions during the transition from normal cells to cancerous cells does exist, and how to regulate the *in vivo* environment to make the alternating pattern return to the normal state. The principles of the first three categories of issues may apply equally to this category.

## NERVOUS SYSTEM AND BRAIN SCIENCE

The crosscutting interaction of the neurology, brain science, and information science will contribute to the advent of the age of intelligence. The peripheral nervous system acts as a perceptual system and the brain acts as the primary central system for analysing perceptual information. The relationship between the two parts is similar to that of the input, calculation, and output functions of a computing system. Therefore, revealing the multilevel features, especially the mechanisms at each level in processing signals and how different levels relate to each other and transmit information, is the key to cognitive science (that is related to life science but focuses on information). How to make a ‘Yes’ or ‘No’ decision based on countless perceptual information after processing at multiple levels is the core issue. The impact of external conditions on this process and the consequent changes of modes are certainly factors that must be considered from the perspective of a complex system, as mesoscales are the paths to breakthroughs. The process might also show three-regime features, namely, ‘Yes’, ‘No’, and the in-between ‘Yes-No-Alternation’.

## COMMON SCIENTIFIC PRINCIPLES OF COMPLEXITY SCIENCE, ENGINEERING SCIENCE, BIG DATA, AND ARTIFICIAL INTELLIGENCE

All of the above five categories of issues belong to the domain of complex systems. When these problems are solved, empirical evidence will be generated for the common principles of complex systems. In the meantime, the implicit law of big data in engineering is also derived from the complex systems involved and the revelation of this law will provide physical logic for the development of artificial intelligence. The crosscutting convergence of these aspects will greatly expand the human knowledge systems. In other words, the development of artificial intelligence should focus on the mechanisms of human intelligent activities and the logics underlying complex systems (data) with emphasis on the possible commonalities between these two aspects. Breakthroughs in these fields may not only lay the scientific foundation for a new generation of artificial intelligence [3], but also play an important role in exploring future information technologies such as network, computation, and information security of the next generation.

## HIGH-PERFORMANCE COMPUTING PARADIGM BASED ON THE PHYSICAL LOGIC OF NATURAL AND ENGINEERING ISSUES

The breakthrough of the above six categories of issues will help to clarify the common physical logic that the complex world should follow and will also provide a scientific basis for the establishment of a computing paradigm that conforms to the physical logic of the computed object, thus boosting the development of computational science. In return, the logic of computational science should comply with the laws of cognitive science. The key is that a common logic—the logic of complex systems—should be adopted. The establishment of a computing system based on the common logic of the computed objects should be an important direction for future development and the high efficiency of computing systems should be pursued through multilevel and multiscale physical logic and human cognitive laws.

## LAWS OF THE EVOLUTION AND REGULATION OF THE CLIMATE SYSTEM

Handling global climate change needs to understand and regulate the material world on a mega scale. The principles and methods involved in solving the above seven categories of issues are equally helpful to the study of the climate system. In order to achieve major breakthroughs, the concepts of hierarchy, system, environment, and dominant mechanism should be defined and paid attention to, and the structural dynamic changes should be taken into account. For example, the model of climate prediction should be transformed from the average grid into a structured grid, thus leading to desirable forecasting capability. The simple coarse-graining is therefore inadequate. How to build and process structured grids at different levels based on physical mechanisms is a challenge that has yet to be tackled.

## MATHEMATICAL TOOLS TO SOLVE MULTI-OBJECTIVE PROBLEMS

The solution of the above eight categories of issues must rely on effective mathematical tools. On the one hand, the development of mathematics should result in the creation of new theories. On the other hand, it must also solve the existing problems. Developing effective mathematical methods to describe all complex systems is a very important research direction. For example, how to formulate the dynamic changes of complex systems governed by multiple dominant mechanisms, most likely in the form of multi-objective variational problems, is a challenging mathematical issue worthy of great attention. The most difficult step in solving this issue is to transform the multiple objectives into a single objective, either in physical ways or in mathematical ways, to reach an inherently identical solution.

In the governance of social and economic systems, for instance, to achieve the 17 sustainable development goals identified in the UN 2030 Agenda, the proposed research strategy is also applicable, as discussed in [4]. In fact, the common focus in various fields is essentially the mesoscale issues at different levels of the world, as exemplified in the above categories for some levels and in our previous attempts even for the level of quantum and that of the universe [1,2]. Hopefully, application of the proposed strategy can yield new understandings, of course, calling for the joint efforts of different disciplines. Knowledge of different disciplines should not only complement each other, but also provide evidence for identifying the laws of commonality.

These issues encompass a wide range of disciplines, hopefully reflecting the complex issues at various levels in different fields. They might be well representative in fulfilling the proposed research strategy and closely related to current global challenges. They are proposed only as ‘possible strategies in adaption to the paradigm shift’ to inspire new ideas and studies. It is our hope that, together, we can gradually clarify the research strategy under the new scientific paradigm so that the paradigm shift in science can be accelerated, and the solutions to global challenges can be achieved.
